# Diabetic foot infections: how to investigate more efficiently? A retrospective study in a quaternary university center

**DOI:** 10.1186/s13244-022-01228-1

**Published:** 2022-05-10

**Authors:** Aisin Ibrahim, Moncef Berkache, Philippe Morency-Potvin, Daniel Juneau, Martial Koenig, Karine Bourduas, Véronique Freire

**Affiliations:** 1 Department of Diagnostic Radiology, McGill University, 1650 Cedar Avenue (Rm C5-118), Montreal, QC H3G 1A4 Canada; 2 Faculty of Medicine, Laval University, 1050 Avenue de la Médecine, Quebec, QC G1V 0A6 Canada; 3 Department of Microbiology and Infectious Disease, Centre hospitalier de L’Université de Montréal, 1051 Rue Sanguinet, QC H2X 3E4 Montreal, Canada; 4 Department of Nuclear Medicine, Centre hospitalier de L’Université de Montréal, 1051 Rue Sanguinet, QC H2X 3E4 Montreal, Canada; 5 Department of Internal Medecine, Centre hospitalier de L’Université de Montréal, 1051 Rue Sanguinet, QC H2X 3E4 Montreal, Canada; 6 Department of Surgery, Orthopedics Division, Centre hospitalier de L’Université de Montréal, 1051 Rue Sanguinet, QC H2X 3E4 Montreal, Canada; 7 Department of Diagnostic Radiology, Centre hospitalier de L’Université de Montréal, 1051 Rue Sanguinet, QC H2X 3E4 Montreal, Canada

**Keywords:** Diabetic foot, Foot Osteomyelitis, Investigation, Magnetic resonance imaging, Gallium bone scan

## Abstract

**Background:**

Diabetic foot infections are frequent and associated with substantial morbidity and substantial cost to the healthcare system. Up to 34% of diabetic patients will develop an ulcer potentially leading to osteomyelitis. Imaging plays a crucial role in the diagnostic process. Imaging modalities to investigate the diabetic foot infection are many and imaging prescription habits remain heterogeneous across physicians. We aimed to improve the appropriateness of imaging examination requested, and performed, for diabetic foot osteomyelitis and we aimed to reduce the overall imaging-related cost.

**Methods:**

Local committee was created to develop an algorithm for suspected diabetic foot osteomyelitis. Best practices were defined by the local algorithm. The algorithm was shared with our physicians. Pre- and post-intervention analysis was conducted retrospectively. All adult diabetic patients with suspected foot osteomyelitis were included. Adherence to best practices was measured. Statistical analysis with Chi-Square and two tailed unpaired t-test was performed.

**Results:**

Pre-intervention cohort had 223 patients (mean age: 63; 168 men). Adherence to best practice was 43%. Scintigraphy (48%) preferred over MRI (44%) and performed simultaneously in 15 patients. Post-intervention cohort had 73 patients (mean age: 66; 62 men). Adherence to best practice was 78%, improved by 35% (*p* < 0.001). MRI (51%) preferred over scintigraphy (23%) and performed simultaneously in three patients. Scintigraphy examinations decreased by 25% (*p* < 0.001). MRI examinations increased by 7% (*p* = 0.32). Hospital imaging related fees decreased by 22% per patient (*p* = 0.002).

**Conclusion:**

Interval improvement in adequate adherence while reducing unnecessary examinations for patients and decreasing costs for the healthcare system was observed.

## Key points

- Local algorithm improved best practices in diabetic foot osteomyelitis investigation by 35%.
- Local intervention significant decreased scintigraphy use (25%) with mild increase in MRI (7%).
- Investigation algorithm for diabetic foot osteomyelitis reduced imaging-related fees by 22%.

## Background

Diabetic foot pathologies are frequently seen in clinical settings and are associated with substantial morbidity, excess care and substantial cost to the healthcare system [1–3]. Studies have shown that up to 50% of diabetic patients will develop a limb neuropathy and between 19 and 34% of diabetic patients will develop a neuropathic and/or ischemic ulcer in their lifetime [4–6]. Moreover, between 20 and 60% of these ulcers will get infected and can potentially evolve into osteomyelitis [7]. Diabetic foot infections often require prolonged use of broad-spectrum antibiotics that can promote antimicrobial resistance [8]. The healthcare burden of diabetic foot infections is significant as it is the number one cause of non-traumatic lower limb amputation and they present a five-year survival rate of less than 30% [9].

Imaging plays a crucial role in the diagnostic process and contributes to tailoring patient management [10]. Imaging modalities to investigate the diabetic foot infection are many and choices include conventional radiographs, ultrasound, computer tomography, magnetic resonance imaging, bone scintigraphy with or without Gallium or labelled leukocytes *and other imaging modalities.*

In 2008, the American College of Radiology (ACR) published the Appropriateness Criteria for Suspected Diabetic Foot Osteomyelitis, which was further reviewed in 2012 and again in 2019 [11, 12]. Additionally, these criteria were also in concordance with the Infectious Diseases Society of America (IDSA) clinical practice guidelines, published in 2012 [13]. Nonetheless, imaging prescription habits remain heterogeneous across physicians and different healthcare institutions [14–16]. The investigation of diabetic foot should always begin with a physical examination and a conventional radiograph. In the presence of an infected-looking wound or ulcers, an MRI is the examination of choice. A bone scintigraphy with white blood cells (WBC) scanning or Gallium scintigraphy is used, when an MRI is contraindicated. It is also recommended that physicians should avoid requesting or performing an MRI and a bone scintigraphy at the same time, and rather only do so in the event of a diagnostic impasse. These practices are expensive and go against the spirit of efficiency and relevance that the healthcare system is currently seeking.

## Materials and methods

We initiated this study to improve the appropriateness of imaging examination requested and performed by the physicians in our institution. Accordingly, our goal was also to improve the tailoring of patient management, to reduce needless simultaneous imaging examinations, and to decrease the overall cost to the healthcare system.

This is a retrospective study that consists of two phases where adherence to best practices was measured before and after an active intervention. The study was approved by the local ethics committee and informed consent was waived due to the retrospective nature. The study was performed at a university-based hospital, the Centre hospitalier de l’Université de Montréal.

### Committee and algorithm creation

**Fig. 1:**
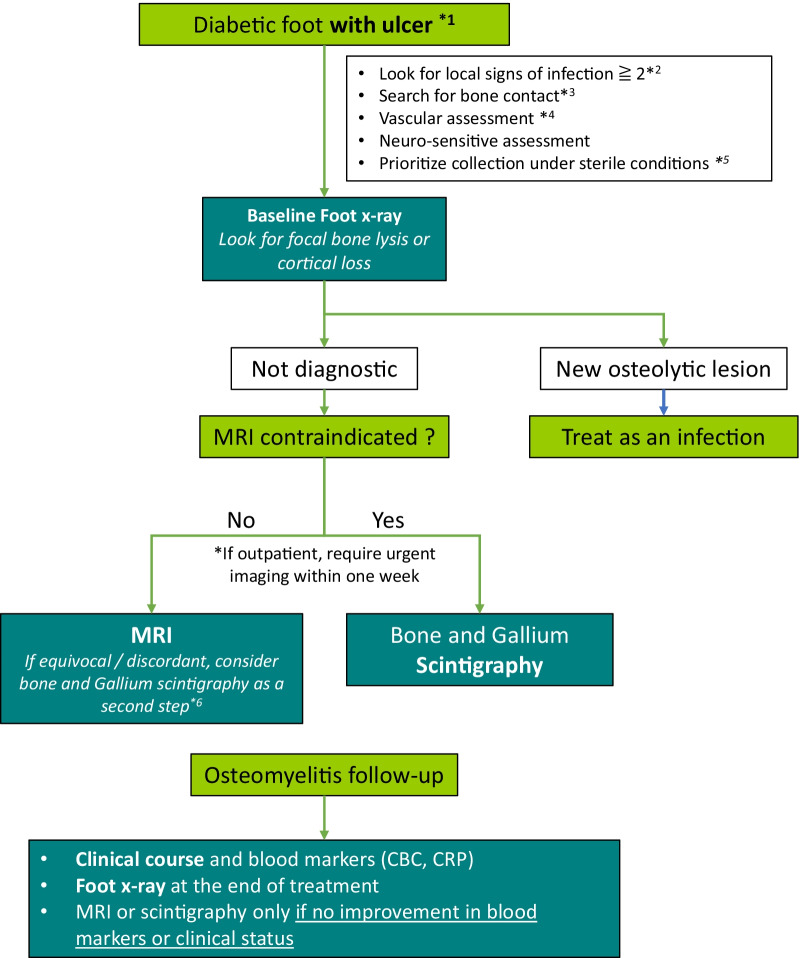
Proposed diabetic foot investigation algorithm. *1: if no ulcer/orifice, osteomyelitis is unlikely and consider another diagnosis and scintigraphy *2: according to IDSA: swelling, redness, pain, heat or purulent discharge *3: bone exposure or bone contact with the metal probe *4: consider arterial Doppler or consultation in vascular surgery if pulse absent / decreased *5: Superficial swabs are not recommended *6: Scintigraphy could be an alternative to first-line MRI

Best practice in terms of adequate use of imaging modalities for suspected diabetic foot osteomyelitis was defined by concordance with a local algorithm. A local committee of multidisciplinary expert physicians was created in early 2019 to develop the algorithm. The committee consisted of a musculoskeletal radiologist, an infectious disease specialist, a nuclear medicine specialist, an internist and an orthopedic surgeon. The expert committee reviewed the American College of Radiology Appropriateness Criteria and the latest literature available on osteomyelitis in the context of a diabetic foot. Using a Delphi consensus method, four rounds of discussion were performed between June and August 2019, in order to establish a local algorithm for investigating diabetic foot osteomyelitis [17]. The final consensus algorithm is shown in Fig. 1.

### Data collection and statistical analysis

Phase 1 of our study occurred before the implementation of the algorithm, between January 1, 2016, and December 31, 2017, while phase 2 occurred after the implementation, between November 1, 2019, and June 30, 2020. Data were collected retrospectively in both phases.

All adult patients with diabetes mellitus presenting with a suspected osteomyelitis located at the level of the foot or ankle were included. Exclusion criteria included: under 18 years of age, pregnancy, and the absence of radiological investigations at the time of presentation. Non-diabetic patients and those with osteomyelitis located elsewhere than at the level of the foot or ankle (e.g., sacrum), and recurrent foot osteomyelitis were also excluded. Medical files and radiological investigations were then reviewed. Our collected data included if the presence of foot ulcers was documented, and the date and type of the imaging performed during the course of treatment. Standard practice in our institution was then compared to the local algorithm to evaluate adherence to best practice.

The algorithm was implemented in September 2019, and it was presented to clinicians in our institution. Initially, we presented the algorithm by means of an oral presentation in Internal Medicine Grand Rounds on September 18, 2019. We had approximately 60 prescribers, including residents and attending physicians, present on site and by videoconference. Subsequently, the algorithm was shared with the physicians by email (approximately 350 recipients). The targeted departments included internal medicine, infectious disease, orthopedic surgery, nuclear medicine and radiology.

**Fig. 2:**
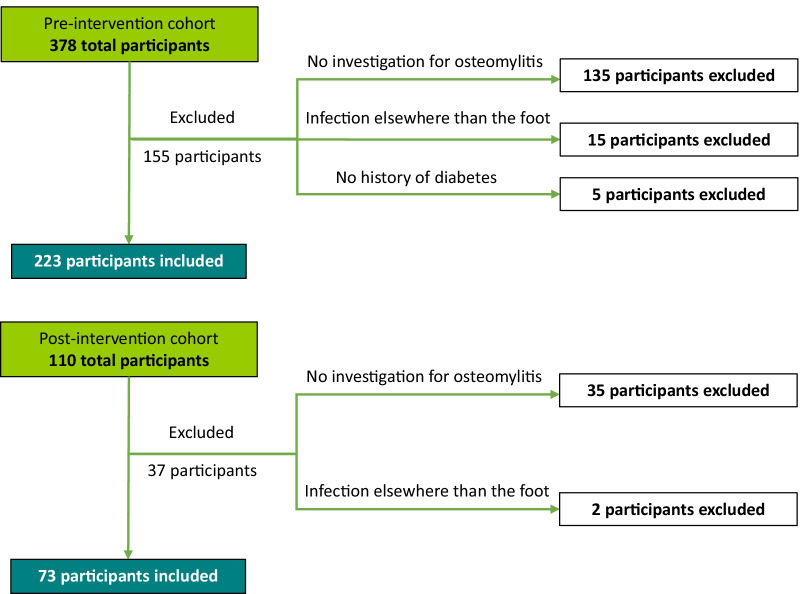
Participants flow diagram for pre- and post-intervention cohorts

In phase 2, we used the same inclusion and exclusion criteria as phase 1. A simplified evaluation grid was used to establish post-intervention adherence to the local algorithm following the best practice standards. Sample size calculation for the phase 2 was based on the precision of the confidence interval for a proportion. We anticipated an increase to 70% adequate adherence after our intervention and wanted to evaluate this proportion with a 10% margin of error. Using a two-sided confidence interval with a confidence level of 0.95, the required number of patients were estimated at *N* = 81 to reach the appropriate level of precision for the confidence interval. Then, statistical analysis was performed using StatView software (SAS Institute, Cary, NC) for the calculation of the Chi-Square to compare the demographic characteristics and examination performed in the two cohorts; and a two-tailed unpaired t-test was performed, to compare the hospital imaging-related fees for both cohorts. Statistical significance was defined as *p* < 0.05 (Fig. 2).

## Results

In phase 1, 378 participants were screened with a total of 223 patients included in the first cohort (168 males and 55 females, mean age: 63 years old). An initial foot radiograph was obtained 84% of the time (186 patients). An MRI was requested and performed in 98 patients (44%) with a mean time of 5.5 days (4 days median). Combined bone scan and Gallium scintigraphy was preferred by the clinicians over MRI and was performed in 108 patients (48%) with a mean time of 5.7 days (4 days median) for bone scan plus an additional delay of 24 to 72 h for Gallium scintigraphy. A total of 15 patients (7%) had both examinations requested and performed simultaneously. Adherence to the algorithm was seen in 96 patients (43% [95% CI: 37, 50]).

In phase 2, 110 participants were screened with a total of 73 patients included in the second cohort. This allowed us to reach a precision of 11.3%, which we considered adequate for the purpose of the study. The post-intervention cohort had similar demographic parameters to the pre-intervention cohort (62 males and 11 females, mean age: 66 years old). An initial foot radiograph was obtained 89% of the time (65 patients). An MRI was performed in 37 patients (51%) with a mean time of 3.5 days (2 days median). Combined bone scan and Gallium scintigraphy was performed in 17 patients (23%) with a mean time of 3.5 days (3 days median) plus an additional delay of 24 to 72 h for Gallium scintigraphy. A total of three patients (4%) had both examinations requested and performed simultaneously. Adherence of the second cohort to the local investigation algorithm was seen in 57 patients (78% [95% CI: 62, 83]) with a significant improvement of 35% *X*^2^ (1, *N* = 296) = 19,7, *p* < 0.001. Demographic and comparative data of both cohorts are summarized in Tables 1 and 2.

**Table 1:** Patient demographic characteristics for the pre- and post-intervention cohorts

	Total	Pre-intervention cohort (*n* = 223)	Post-intervention cohort (*n* = 73)	*p* value
Male (%)	230 (77)	168 (75)	62 (85)	*p* = 0.06
Age (mean ± SD)	64 ± 11.78	63 ± 12.14	66 ± 10.46	*p* = 0.10
Foot ulceration (%)	279 (94)	214 (95)	66 (90)	*p* = 0.13

**Table 2:** Comparative data for the pre- and post-intervention cohorts

	Pre-intervention cohort (*n* = 223)	Post-intervention Cohort (*n* = 73)	*p* value
Adherence	43% (96)	79% (58)	*p* < 0.001
Foot radiograph	84% (186)	89% (65)	*p* = 0.17
Foot MRI	44% (98)	51% (37)	*p* = 0.32
Foot scintigraphy	48% (108)	23% (17)	*p* < 0.001
Dual examination	7% (15)	4% (3)	*p* = 0.36

Interestingly, there was a significant decrease in scintigraphy examination performed by 25%, *X*^2^ (1, *N* = 296) = 14,25, *p* < 0.001 versus a slight, non-statistically significant increase in MRI requests by 7%, *X*^2^ (1, *N* = 296) = 1,00, *p* = 0.32. These changes led to an average decrease in the hospital imaging related fees of 83,95*$CAD per patient, which represents a decrease of 22% (*p* = 0.002, two-tailed unpaired t-test). A detailed comparison of these fees is presented in Table 3.

**Table 3:** Comparison between the hospital imaging related fees for the pre- and post-intervention cohorts

	Patient (#)	Fees ($CAD)^4^	Total ($CAD)
*Pre-intervention cohort*			
Foot MRI^1^	98	335.00	32,830.00
Foot scintigraphy^2^	108	429.80	46,418.40
Foot radiograph^3^	186	27.27	5072.22
Total hospital fees	223	–	84,320.62
Average hospital fee per patient	–	–	378.12
*Post-intervention cohort*			
Foot MRI^1^	37	335.00	12,395.00
Foot scintigraphy^2^	17	429.80	7306.60
Foot radiograph^3^	65	27.27	1772.55
Total hospital fees	73	–	21,474.15
Average hospital fee per patient	–	–	294.17

^1^Foot MRI; our standard MRI protocols are usually performed on a 3 T imaging system with a dedicated foot and ankle coil and include: coronal and axial fat-saturated FSE T2-weighted images (Echo time (TE) 80–100, repetition time (TR) 2800–8000, echo train length (ETL) 11–13), coronal and sagittal FSE T1-weighted (TE 10–30, TR 450–750, ETL 3–5) and if creatinine clearance allows it, coronal and sagittal fat-saturated T1-weighted imaging following IV gadolinium administration

^2^Foot scintigraphy; our local protocol always includes a tri-phasic planar bone scan, with the addition of SPECT-CT acquisition when pertinent. This is then followed by planar ± SPECT-CT Gallium scintigraphy, when pertinent. This results in a total fee that can range from 214$CAD to 614$CAD, with an average fee of 429,80$CAD

^3^Foot radiograph; includes a standard AP, lateral and oblique views of the foot

^4^The fees are mandated by Québec’s Health and Social services Ministry and can be found on the ministry website. These fees are related to manpower and equipment related to imaging in the hospital setting, they do not include the radiologist or nuclear medicine specialist professional fee [20]

## Discussion

The aim of this study was to improve the appropriateness of imaging requested and performed for diabetic foot osteomyelitis by physicians in our institution. We aimed to reduce needless simultaneous imaging examinations and to decrease the overall cost to the healthcare system. This study demonstrates that this is achievable in an uncomplicated manner, even in a large university-based healthcare center with two distinct Radiology and Nuclear Medicine departments.

Our study specifically targeted diabetic foot osteomyelitis, as it is a significant cause of mortality and morbidity. It also represents a significant cost to the healthcare system, where imaging plays a crucial role in the diagnosis and contributes to tailoring patient management.

Following the intervention, nuclear medicine examinations, mostly bone scan and Gallium scintigraphy, have significantly decreased by 25%. On the other hand, MRI examinations were only increased by 7%. We were able to achieve a post-intervention adherence of 78% compared to our pre-intervention cohort which was 43%, therefore increasing the adequate adherence to our algorithm by 35% (*p* < 0.001). This intervention has reinforced the adherence to the ACR and IDSA appropriateness criteria, emphasizing the use of MRI rather than the use of nuclear medicine examinations as a first-line investigation for suspected foot osteomyelitis in diabetic patients with pedal ulcers. In a recent metanalysis, Llewellyn et al. reported a higher diagnostic accuracy with MRI (95.6% sensitivity; 80.7% specificity) when compared to conventional scintigraphy examinations (83.6% sensitivity; 70.6% specificity). However, WBC scintigraphy had a higher diagnostic accuracy (87.3% sensitivity; 94.7% specificity), similar to MRI. Therefore, can be osteomyelitis reliably diagnosed by MRI and WBC scintigraphy. Nonetheless, the wider availability of MRI machines, the lack of exposure to ionizing radiation, the higher spatial resolution allowing surgical planning if needed, and the higher costs related to WBC scintigraphy make MRI the preferred modality in most cases [18]. In the absence of a foot ulcer, however, nuclear medicine examinations such as ^18^F-FDG PET/CT may be appropriate, as per the ACR appropriateness criteria, in diagnosing deep soft-tissue infection and differentiating osteomyelitis and from Charcot neuro-arthropathy, especially for patients with metallic implants, for whom MRI examination would be limited [12]. Interestingly, a recent study by Diez et al. has shown that ^18^F-FDG PET/CT was the most accurate technique for differentiation of Charcot neuro-arthropathy from diabetic foot OM [19]. To our knowledge, this is the first study that focuses specifically on the application of the appropriateness criteria, whereas other studies have focused on the diagnostic hallmarks of osteomyelitis.

During our meeting with the clinicians, they reported a subjective impression of MRI inaccessibility due to limited access in the past, as well as the cost, and time constraints. Their impression could be used as a hypothesis to explain the initial underutilization of MRI versus nuclear medicine examinations. This could also explain the simultaneous request of different imaging modalities with the intent to expedite a diagnosis for patient management. However, our study demonstrated that the timing between an entered requisition and execution was similar for an MRI (mean: 3.5 days, median 2 days) and for scintigraphy (mean: 3.5 days, median 3 days) in the post-intervention cohort. Additionally, we noted a decrease from 7 to 4% in the number of simultaneous requested and performed MRI and scintigraphy. Although not statistically significant (*p* = 0.36), the decrease in the needless simultaneous examinations allows for cost and time reduction. Thus, our study has demonstrated that the time of request completion should not be a factor that motives requesting multiple examinations simultaneously.

Furthermore, to assess the repercussions of our intervention, we assessed how our findings impacted the overall cost to the healthcare system. There was a 22% (*p* = 0.002) net decrease in hospital imaging-associated fees per patient. In public healthcare systems, this is a major factor that allows for a more appropriate fund allocation and improving cost-effectiveness.

There are a few limitations to our study. The first limitation is related to the retrospective nature of the study. Although most of the data was collected on our PACS system, some patients may have had imaging or follow-up imaging performed in other institutions, which would not have been available to review. Moreover, we had a smaller post-intervention cohort with 73 patients for an 8-month period versus 223 patients over a 24-month period in the pre-intervention cohort. However, our cohorts did not significantly differ in demographic constitution and results were found to be statistically significant.

Another limitation is that our algorithm includes bone scintigraphy with Gallium scintigraphy, as opposed to WBC scintigraphy, as is generally recommended in the literature. This is due to our local reality and expertise. WBC tagging is not performed locally in our institution, therefore the blood samples need to be transported to another facility and then returned in order to be injected into the patient. This implies significant costs, and limited slots are available. In contrast, the local nuclear medicine team has extensive experience and a strong expertise in the interpretation of Gallium examinations. It should also be noted that most studies investigating the accuracy of Gallium scintigraphy in OM are older, and most used only planar images. In contrast, the local practice involves triple-phase bone scan, with planar ± SPECT-CT acquisitions, followed when required by Gallium scintigraphy with planar ± SPECT-CT acquisitions. WBC scintigraphy is reserved for equivocal or very complex cases.

As a confounding factor, the post-intervention data acquisition was performed between November 1, 2019, and June 30, 2020, during the COVID-19 pandemic. However, we estimate that this factor did not play a significant role as our study targeted only emergency patients and not out-patients. These patients were mostly treated by the same doctors as the pre-intervention cohort and both radiological examinations were as available as in the pre-pandemic cohort.

The validity of our results could potentially be strengthened by a longer post-intervention follow-up. It would be particularly interesting to explore what will happen two to three years after the intervention, when new clinicians, residents or attendings, join our institution. This assessment will be the true measure of whether we managed to implant a long-lasting change in diabetic foot osteomyelitis investigation.

## Conclusion

Our intervention has succeeded to improve the rate of adherence to best practices for imaging of suspected diabetic foot osteomyelitis by 35% following implementation of our algorithm. This reduced the number of scintigraphy examinations by 25% without a significant increase in the number of MRI scans (7%). This led to an overall 22% decrease in imaging-related fees.

## Data Availability

The datasets used and/or analyzed during the current study are available from the corresponding author on reasonable request.

## Abbreviations

ACR: American college of radiology
IDSA: Infectious diseases society of America
MRI: Magnetic resonance imaging
SPECT-CT: Single-photon emission computerized tomography
WBC: White blood cell
